# Specnuezhenide suppresses diabetes-induced bone loss by inhibiting RANKL-induced osteoclastogenesis: Specnuezhenide suppresses diabetes-induced bone loss

**DOI:** 10.3724/abbs.2022094

**Published:** 2022-08-04

**Authors:** Xiaoshuang Ye, Juanjuan Jiang, Juan Yang, Wenyan Yan, Luyue Jiang, Yan Chen

**Affiliations:** Department of Nephrology the Affiliated Geriatric Hospital of Nanjing Medical University Nanjing 210024 China

**Keywords:** specnuezhenide, osteoclastogenesis, bone loss, RANKL, NF-κB, MAPK

## Abstract

Diabetes osteoporosis is a chronic complication of diabetes mellitus (DM) and is associated with osteoclast formation and enhanced bone resorption. Specnuezhenide (SPN) is an active compound with anti-inflammatory and immunomodulatory properties. However, the roles of SPN in diabetic osteoporosis remain unknown. In this study, primary bone marrow macrophages (BMMs) were pretreated with SPN and were stimulated with receptor activator of nuclear factor kappa B ligand (RANKL; 50 ng/mL) to induce osteoclastogenesis. The number of osteoclasts was detected by tartrate-resistant acid phosphatase (TRAP) staining. The protein levels of cellular oncogene fos/nuclear factor of activated T cells c1 (c-Fos/NFATc1), nuclear factor kappa-B (NF-κB), and mitogen-activated protein kinases (MAPKs) were evaluated by western blot analysis. NF-κB luciferase assays were used to examine the role of SPN in NF-κB activation. The DM model group received a high-glucose, high-fat diet and was then intraperitoneally injected with streptozotocin (STZ). Micro-CT scanning, serum biochemical analysis, histological analysis were used to assess bone loss. We found that SPN suppressed RANKL-induced osteoclast formation and that SPN inhibited the expression of osteoclast-related genes and *c-Fos*/ *NFATc1*. SPN inhibited RANKL-induced activation of NF-κB and MAPKs. *In vivo* experiments revealed that SPN suppressed diabetes-induced bone loss and the number of osteoclasts. Furthermore, SPN decreased the levels of bone turnover markers and increased the levels of runt-related transcription factor 2 (RUNX2), osteoprotegerin (OPG), calcium (Ca) and phosphorus (P). SPN also regulated diabetes-related markers. This study suggests that SPN suppresses diabetes-induced bone loss by inhibiting RANKL-induced osteoclastogenesis, and provides an experimental basis for the treatment of diabetic osteoporosis.

## Introduction

Diabetes osteoporosis is a chronic complication of diabetes mellitus (DM), and is generally caused by an absolute or relative deficiency of insulin, a hormonal imbalance caused by endocrine dysfunction, or calcium (Ca) and phosphorus (P) metabolism disorders, resulting in decreased bone density and changes in bone microstructure [1]. Recently, an increasing number of studies have noted that diabetes has a negative impact on bone, and the risk of fracture in patients can be up to 1–3 times more likely. Osteoporosis is characterized by decreased bone mass and bone strength [ 2– 5] . The major pathological feature of osteoporosis is the increased osteoclast formation and activation [6]. Pathologically over-activated osteoclasts promote bone resorption and inhibit bone formation by inhibiting the activity of osteoblasts, leading to progressive bone loss in osteoporosis [ 7, 8] . Therefore, osteoclasts are the main target of drugs for the treatment of osteoporosis.

As a classic inducer of osteoclast formation, receptor activator of nuclear factor kappa B ligand (RANKL) plays a decisive role in osteoclast formation, activation and survival [ 9, 10] . The interaction between RANKL and its receptor RANK in osteoclast precursor cells triggers a series of signaling pathways, including mitogen-activated protein kinases (MAPKs), nuclear factor kappa-B (NF-κB), activator protein 1 (AP-1), and nuclear factor of activated T cells c1 (NFATc1) [ 11– 13] , among which the NF-κB pathway and MAPK pathway are the two most important signaling pathways in the process of osteoclast formation [ 14, 15] . Studies have shown that mice with MAPK-related gene deletion can present with osteoclast formation disorders and a higher risk of osteosclerosis [ 16, 17] . In addition, the NF-κB signaling pathway is also an important research target for the treatment of osteoporosis, and molecules in the pathway, including protein phosphorylation and nuclear translocation, may become drug targets [ 18, 19] .

**Figure 1:**
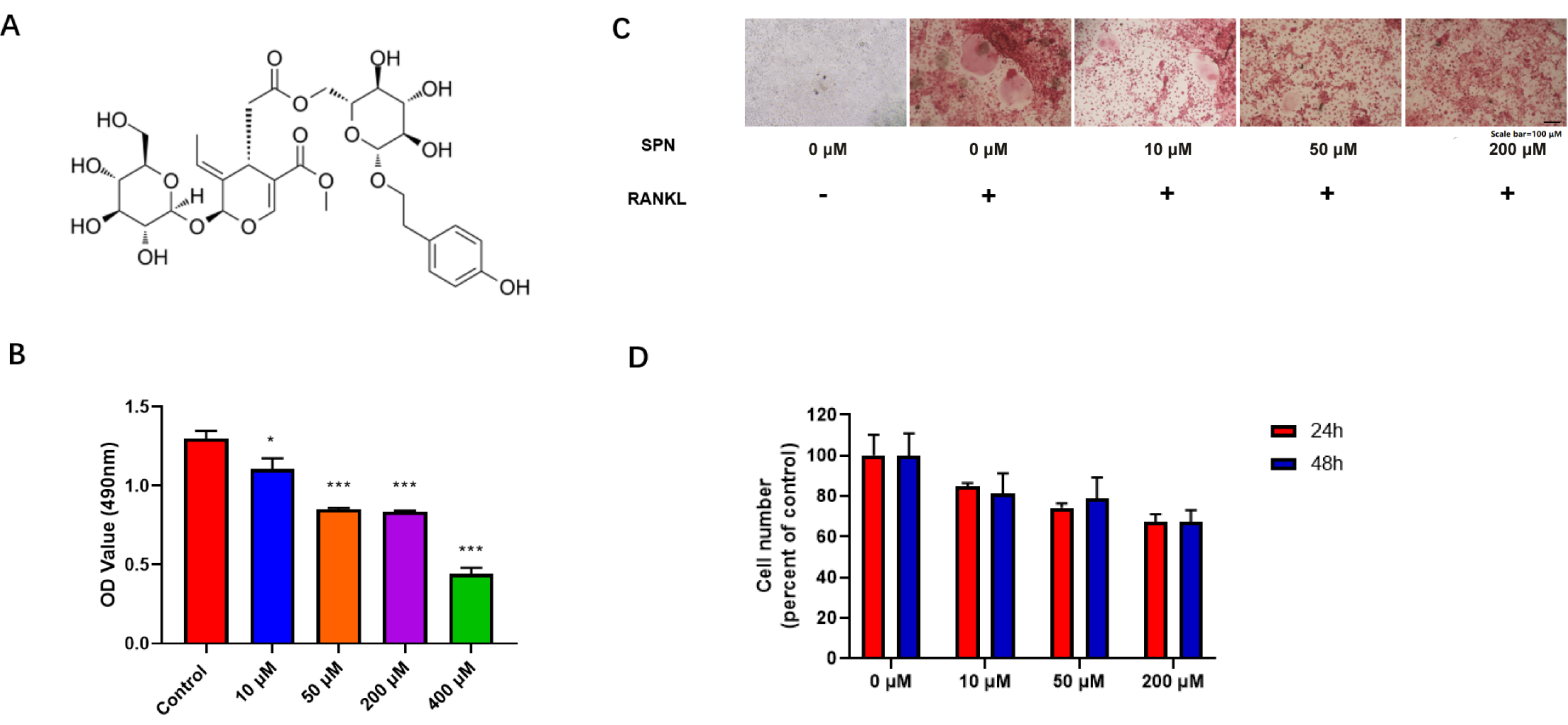
Figure 1 Specnuezhenide inhibits RANKL-induced osteoclastogenesis (A) Chemical structure of SPN. (B) MTT assays were performed after incubation of BMM cells with SPN (0, 10, 50, 200, and 400 μM) for 24 h and 48 h at 37°C in a 5% CO 2 atmosphere. (C) BMMs were plated into 96-well plates (0.32 cm 2) at 1×10 4 cells/well, and pretreated with different concentrations of SPN (0, 10, 50 and 200 μM) for 30 min and incubated with RANKL (50 ng/mL) for 3 days. Osteoclastic formation from BMMs was examined by staining for TRAP activity and is indicated by black arrows. Scale bar= 100 μm. (D) The number of osteoclasts in 96-well plates (0.32 cm 2) was counted. Data are expressed as the mean±SD of triplicate experiments. * P<0.05, *** P<0.001 vs RANKL-treated control.

In recent years, there have been an increasing number of studies on the key signaling pathways and cytokines extracted from traditional Chinese medicine to regulate the formation and function of osteoclasts, which has also brought new options for the treatment of osteoporosis [20]. Specnuezhenide (SPN; C _31_H _42_O _17_, molecular weight 686.62; Figure 1A) is an active constituent of soluble fissured cycloiridoid glycosides in *Ligustrum* [21]. Studies have shown that SPN has inhibitory effects on MAPK/extracellular regulated protein kinases (ERK), hypoxia-inducible factor 1-alpha/vascular endothelial growth factor (HIF-1α/VEGF) and other inflammation-related signaling pathways and has anti-inflammatory, immunomodulatory and anti-aging properties [ 22– 24] . Due to the recent discovery of SPN, there are few studies on it, and its medicinal value is not clear [23]. The anti-inflammatory and immunomodulatory properties of SPN may justify its role in osteolytic bone disease. However, to date, the direct effect of SPN on diabetes-induced osteoporosis has not been investigated.

Streptozotocin (STZ) is an antibiotic extracted from *Streptomyces sp* [25]. STZ can specifically destroy islet β cells, causing autoimmune inflammatory damage and apoptosis of islets [26]. Damage to islet β cells leads to a rapid decrease in insulin secretion and a significant increase in blood sugar, showing the appearance of diabetes [26]. Numerous studies have established a rat model of diabetic osteoporosis by combining a high-glucose, high-fat diet with streptozotocin therapy [ 27, 28] . In this study, we investigated the effect of SPN on osteoclast formation and diabetic osteoporosis, which provided a research basis for the possibility of natural compounds as alternative drugs for the prevention and treatment of osteoporosis.

## Materials and Methods

### Chemicals

SPN (purity>98%) was obtained from Shanghai Pureone Biotechnology (Shanghai, China). A luciferase assay system was obtained from Promega (Madison, USA). RANKL was obtained from Sigma (St Louis, USA). Primary antibodies and secondary antibodies were obtained from Santa Cruz Biotechnology (Santa Cruz, USA). Fetal bovine serum (FBS) and alpha-modified Eagle’s medium (α-MEM) were purchased from Gibco BRL (Grand Island, USA). Recombinant macrophage-colony stimulating factor (M-CSF) and RANKL were obtained from R&D Systems (Minneapolis, USA). Other chemicals were purchased from Sigma.

### Osteoclastogenesis *in vitro*

Primary bone marrow macrophages (BMMs) were isolated from the femur and tibia of male C57BL/6 mice (6–8 weeks old) as described previously [29]. BMMs were cultured in α-MEM supplemented with 10% FBS, 1% antibiotics (100 U/mL penicillin and streptomycin) and 20 ng/mL M-CSF at 37°C with 5% CO _2_. To explore the role of SPN in BMM osteoclastogenesis, BMMs were plated into 96-well plates (0.32 cm ^2^) at 1×10 ^4^ cells/well. Then, BMMs were pretreated with various concentrations of SPN (0, 10, 50, and 200 μM) for 30 min and cultured with 50 ng/mL RANKL for 5 days. The medium was replaced every 2 days. After 5 days, the cells were fixed with 4% paraformaldehyde, and tartrate-resistant acid phosphatase (TRAP) staining was performed at 37°C. TRAP-positive cells containing ≥3 nuclei were considered osteoclasts and counted using ImageJ software (National Institutes of Health, Bethesda, USA).

### Cell viability assay

Cell viability was measured using the 3-(4,5)-dimethylthiahiazo(-z-y1)-3,5-di-phenytetrazoliumromide (MTT) method as previously described [30]. Briefly, BMMs were plated in 96-well plates with SPN (0, 10, 50, 200, or 400 μM) at a density of 1×10 ^3^ cells/well and cultured with complete α-MEM and M-CSF. After 24 and 48 h, the viability of BMMs was determined via MTT assays.

### RNA extraction and quantitative real-time PCR

Quantitative real-time PCR (qRT-PCR) was used to measure the expressions of specific genes during osteoclast formation. To investigate whether SPN regulates the expression of osteoclast-associated genes in a dose-dependent manner, BMMs were pretreated with SPN at concentrations of 0, 50, and 200 μM for 30 min and stimulated with RANKL for 5 days. In addition, to investigate whether SPN regulated the expressions of osteoclast-associated genes in a time-dependent manner, BMMs were pretreated with 200 μM SPN for 30 min and stimulated with RANKL for 0, 1, 3, and 5 days. Total RNA was isolated with TRIzol reagent (Invitrogen, Carlsbad, USA) according to the manufacturer’s instructions. Total RNA was used for reverse transcription to synthesize cDNA with a cDNA Synthesis Kit (Thermo Fischer Scientific, Waltham, USA). qRT-PCR was performed using SYBR Premix Ex Taq II (Takara, Tokyo, Japan) with an Applied Biosystems 7300 Real-Time PCR System (Applied Biosystems, Foster City, USA). β-Actin was used as an internal control for mRNA. The primers used in this study are listed in Table 1.

**Table:** **Table 1** Sequence of primers used in RT-PCR

Gene	Forward primer (5′→3′)	Reversed primer (5′→3′)
*TRAP*	TCCCCAATGCCCCATTC	CGGTTCTGGCGATCTCTTTG
Cathepsin K	CCAGTGGGAGCTATGGAAGA	AAGTGGTTCATGGCCAGTTC
*DC-STAMP*	CTTCCGTGGGCCAGAAGTT	AGGCCAGTGCTGACTAGGATGA
*c-Fos*	CTGTCAACACACAGGACTTTT	AGGAGATAGCTGCTCTACTTTG
*MMP-9*	GCAAACCCTGCGTATTTCCAT	GATAACCATCCGAGCGACCTTT
*NFATc1*	ACCACCTTTCCGCAACCA	TTCCGTTTCCCGTTGCA
*β‐actin*	ATCACCATTGGCAATGAGCG	TTGAAGGTAGTTTCGTGGAT

### Western blot analysis

To explore the role of SPN in RANKL-mediated expressions of the cellular oncogenes fos (c-Fos) and NFATc1, BMMs were pretreated with 200 μM SPN for 30 min and stimulated with RANKL for 0, 1, 3, and 5 days. To explore the role of SPN on RANKL-mediated expression of NF-κB activation and MAPK activation, BMMs were pretreated with 200 μM SPN for 30 min and stimulated with RANKL for 0, 5, 10, 20, 30, and 60 min. Total protein was extracted using radioimmunoprecipitation assay (RIPA) lysis buffer (Beyotime Biotechnology, Haimen, China). Proteins were separated by SDS-PAGE and then transferred to polyvinylidene difluoride (PVDF) membranes (Merck Millipore, Billerica, USA). After being blocked with 5% skim milk for 1 h at room temperature, the membranes were incubated with the primary antibodies and then with secondary antibodies (Sanying, Wuhan, China). The membranes were developed and signals were detected using an enhanced chemiluminescence kit (Merck Millipore). The antibodies used in this study are as follows: c-Fos (1:500 dilution); NFATc1 (1:500 dilution); NF-κB inhibitor alpha (IkB-α) (1:500 dilution); phosphorylated p38 (p-p38) (1:500 dilution); p38 (1:100 dilution); phosphorylated c-jun n-terminal kinase (p-jnk) (1:1000 dilution); jnk (1:100 dilution); phosphorylated extracellular signal-regulated kinase (p-erk) (1:500 dilution); erk (1:1000 dilution); glyceraldehyde-3-phosphate dehydrogenase (GAPDH) (1:1000 dilution); β-actin (1:1000 dilution); and horseradish peroxidase (HRP)-conjugated mouse anti-rabbit IgG (1:5000 dilution).

### NF-κB luciferase assay

To examine NF-κB activation, BMMs were stably transfected with an NF-κB luciferase reporter constructed as described previously [31]. Briefly, BMMs were seeded in 24-well plates and pretreated with SPN (0, 50, or 200 μM) for 30 min, followed by stimulation with 50 ng/mL RANKL for 6 h (for the NF-κB luciferase reporter gene) [32]. Luciferase activity was measured using the Promega Luciferase Assay System (Promega, Madison, USA) according to the manufacturer’s instructions.

### Establishment of the diabetes osteoporosis rat model

Eight-week-old male Sprague-Dawley (SD) rats were obtained from GemPharmatech Co., Ltd (Nanjing, China). A total of 24 SD rats were randomly divided into 4 groups: the control group; the DM model group; the DM model +50 μM SPN (0.035 mg/kg) group; and the DM model +200 μM SPN (0.14 mg/kg) group. The DM model group received a high-glucose, high-fat diet for five weeks, and was then intraperitoneally injected with STZ (60 mg/kg). After one week, a diagnosis of diabetes was made based on blood glucose (fasting) >11.1 mM. For the SPN-treatment group, 50 μL of SPN solution dissolved in saline (50 μM, 0.035 mg/kg) or 200 μL of SPN solution dissolved in saline (200 μM, 0.14 mg/kg) were injected intraarticularly into DM model rats every 7 days. After 6 weeks of treatment, the rats were sacrificed, and their femurs were collected for follow-up experiments. This *in vivo* experiment was approved by the Affiliated Geriatric Hospital of Nanjing Medical University [Permit number SYXK(SU)2018-0020].

### Micro-CT scanning

The fixed femurs were scanned using high-resolution microcomputed tomography analysis (70 kVp, 110 μA, and 9 μm; Bruker, Kontich, Belgium). Three-dimensional (3D) reconstructions of the femoral structure were performed after scanning. The region of interest (ROI) was generated from the region 5 mm above the growth plate on the distal femur with a height of 1 mm. Then, a constant trabecular binarization threshold was used to assess the bone parameters in the ROI, bone volume/total volume (BV/TV; %), trabecular separation (Tb.Sp, 1/mm), trabecular thickness (Tb.Th, 1/μm), and trabecular number (Tb.N, 1/μm) were used to assess bone loss using CT Analyzer software.

### Serum biochemical analysis

After 6 weeks of treatment, the rats were sacrificed by excessive isoflurane. Blood was collected and centrifuged for 15 min to isolate serum. The levels of diabetes-related markers, including glucose, insulin, hemoglobin A1c (HbA1c), low-density lipoprotein (LDL-C), total cholesterol (TC), triglycerides (TGs), and high-density lipoprotein cholesterol (HDL-c), bone turnover markers, including osteocalcin, ALP, tartrate-resistant acid phosphatase 5b (TRAP-5b), procollagen I N-terminal peptide (PINP), and carboxy-terminal cross-linked telopeptide of type 1 collagen (CTX-1), and bone formation-related markers, including runt-related transcription factor 2 (RUNX 2), osteoprotegerin (OPG), Ca, P, and RANKL, were detected by enzyme-linked immunosorbent assay using the corresponding kits and reagents obtained from Nanjing Jiancheng Bioengineering Institute (Nanjing, China).

### Histological analysis

For histological analysis, the metaphyseal region of femoral bones was fixed in 4% paraformaldehyde for 1 day. After decalcification in 10% ethylenediaminetetraacetic acid (EDTA) for 2 weeks, the sections were embedded in paraffin, sectioned at 5 μm thickness and stained with hematoxylin and eosin (H&E).

### Statistical analysis

All results are presented as the mean±standard deviation (SD) of three experiments. Statistical significance was determined using SPSS software (ver. 22). Significant differences were analyzed by one-way ANOVA or two-tailed Student’s *t*-test. *P*<0.05 was considered statistically significant.

## Results

### Specnuezhenide inhibits RANKL-induced osteoclastogenesis

To explore the role of SPN in RANKL-induced osteoclastogenesis, functional experiments on BMMs are listed in Supplementary Figure S1. To determine whether SPN has toxic effects on BMMs, MTT assay was used to evaluate cell viability. The results showed that even with SPN at 200 μM for 48 h, the cells still maintained a high survival rate ( Figure 1B). However, 400 μM SPN had a slightly toxic effect on BMMs ( Figure 1B). We thus selected 200 μM as the SPN concentration for subsequent experiments. The effects of SPN on RANKL-induced osteoclast formation were first examined. BMMs were pretreated with different concentrations of SPN (0, 10, 50, and 200 μM) and incubated with RANKL for 3 days. After 3 days, the cells were stained with TRAP ( Figure 1C). TRAP-positive cells with more than three nuclei were considered osteoclasts. The results showed that SPN dose-dependently inhibited RANKL-induced osteoclast formation ( Figure 1D). Therefore, we conclude that the inhibition of osteoclast formation by SPN is not induced by its toxicity to mature osteoclasts.

### Specnuezhenide suppresses RANKL-induced gene expression

**Figure 2:**
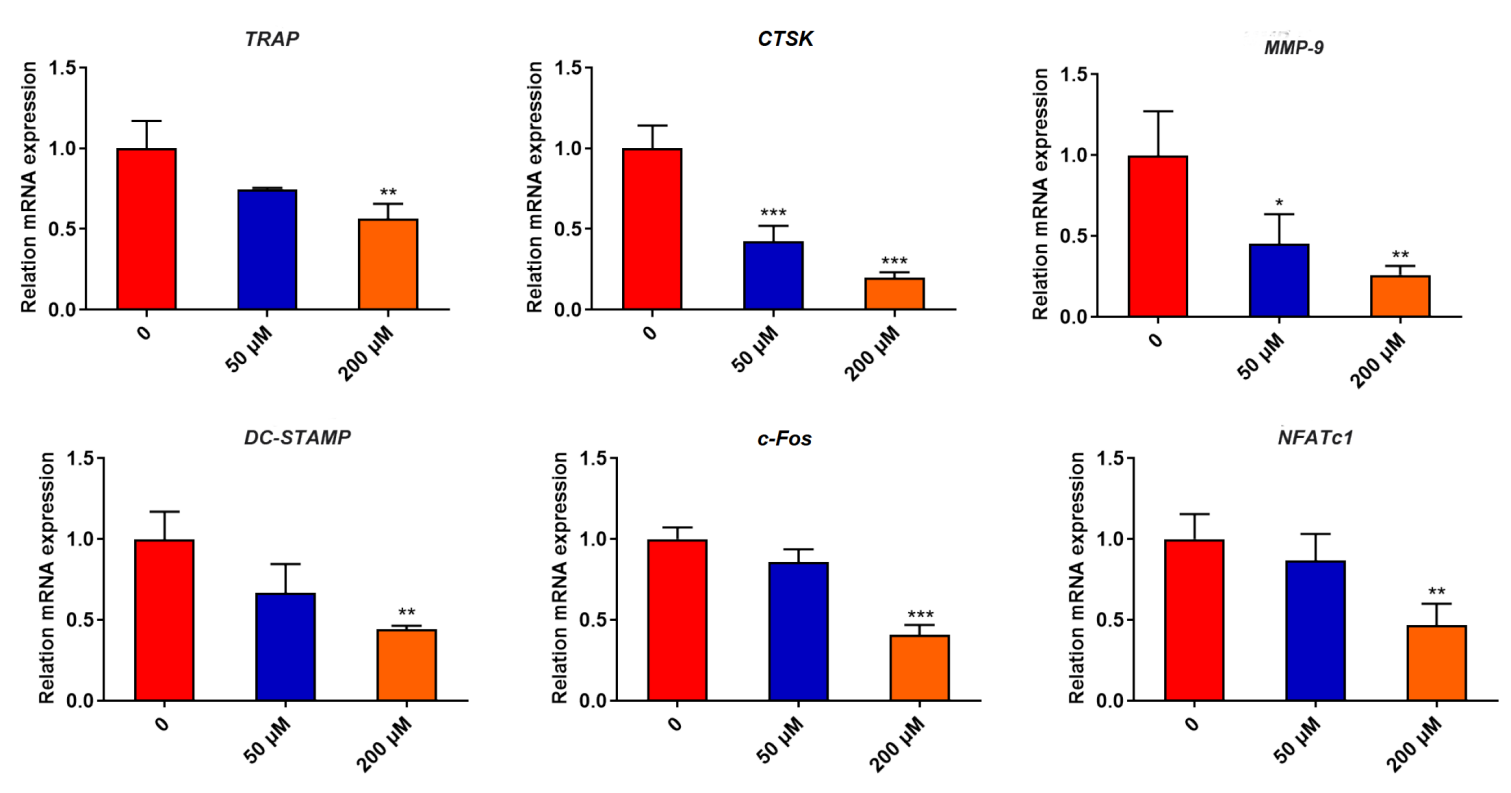
Figure 2 Specnuezhenide dose-dependently reduces RANKL-induced gene expression Total RNA was extracted from BMMs treated with SPN (0, 50 and 200 μM) for 30 min and RANKL (50 ng/mL) for 3 days. Relative gene expressions of TRAP, CTSK, MMP-9, DC-STAMP, c-Fos and NFATc1 were analyzed by qRT-PCR. Data are presented as the mean±SD of three independent experiments. * P<0.05, ** P<0.01, *** P<0.001 vs RANKL-treated control.

nuclear factor of activated T cells 1 (NFATc1) is a key regulator of late osteoclast differentiation and is required for the regulation of many osteoclast-specific genes [ 33, 34] . TRAP, Cathepsin K (CTSK), matrix metalloproteinase-9 (MMP-9), dendritic cell-specific transmembrane protein (DC-STAMP), c-FOS and NFATc1 play important roles in the dissolution of bone matrix [ 35, 36] . Therefore, we used qRT-PCR to examine the effect of SPN on the expressions of osteoclast-associated genes, including *TRAP*, *CTSK*, *MMP-9*, *DC-STAMP*, *c-FOS* and *NFATc1*. To investigate whether SPN regulates the expressions of osteoclast-associated genes in a dose-dependent manner, BMMs were pretreated with SPN at concentrations of 0, 50, and 200 μM for 30 min and stimulated with RANKL for 3 days. As shown in Figure 2, SPN inhibited the expressions of all marker genes in osteoclasts in a dose-dependent manner, consistent with its inhibitory effect on osteoclast formation. These results indicate that SPN suppresses RANKL-induced osteoclast-associated gene expression.

### Specnuezhenide suppresses RANKL-mediated expression of c-Fos/NFATc1

**Figure 3:**
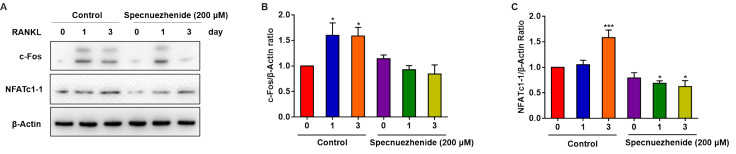
Figure 3 Specnuezhenide inhibits the RANKL-mediated expression of c-Fos/NFATc1 BMMs incubated in serum-free medium for 5 h were pretreated with SPN (200 μM) for 30 min prior to RANKL (50 ng/mL) stimulation for 0, 1, and 3 days. (A–C) Total protein was extracted and subjected to western blot analysis using antibodies against NFATc1 and c-Fos. Data are presented as the mean±SD of three independent experiments. * P<0.05, *** P<0.001 vs RANKL-treated control.

NFATc1 and c-Fos are two crucial transcription factors of osteoclast differentiation that are induced and activated by the RANKL/RANK signaling pathway [ 37, 38] . Therefore, the effect of SPN on NFATc1 and c-Fos protein expression was detected by western blot analysis. BMMs were pretreated with 200 μM SPN for 30 min and stimulated with RANKL for 0, 1, and 3 days. As shown in Figure 3, the protein expressions of NFATc1 and c-Fos were significantly increased during osteoclast generation (1–3 days), whereas 200 μM SPN significantly inhibited their expressions, consistent with previous mRNA levels. Taken together, these results demonstrate that SPN inhibits osteoclastogenesis by downregulating the expressions of c-Fos and NFATc1.

### Specnuezhenide suppresses RANKL-induced NF-κB activation

**Figure 4:**
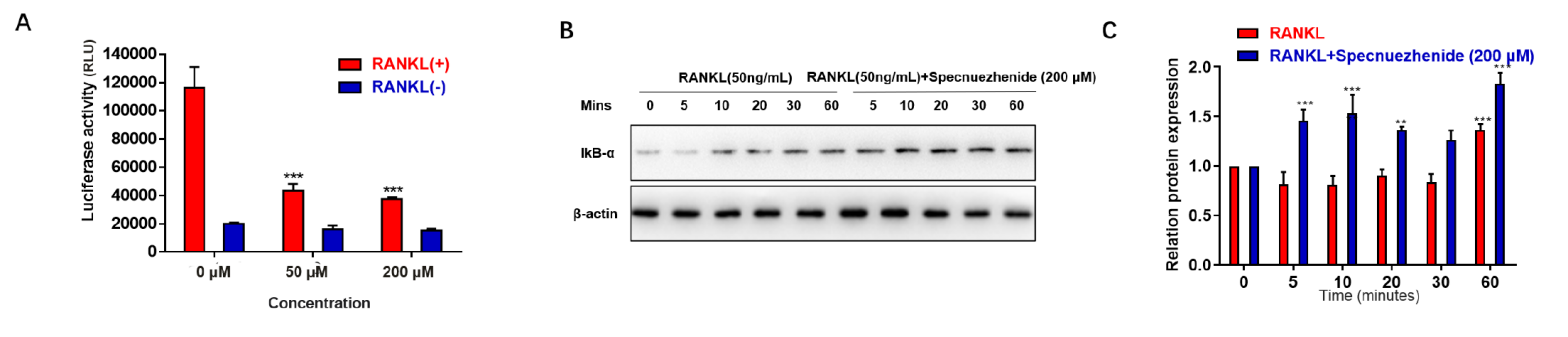
Figure 4 Specnuezhenide suppresses RANKL-induced NF-κB activation (A) BMMs stably transfected with the 3 kB-Luc-SV40 reporter gene were pretreated with SPN (0, 50, and 200 μM) for 30 min, followed by RANKL (50 ng/mL) stimulation for 6 h. Luciferase activity in the lysates was determined after 6 h of RANKL stimulation. (B,C) BMMs were pretreated with SPN (0, 100, and 200 μM) for 30 min, followed by RANKL (50 ng/mL) stimulation for 0, 5, 10, 20, 30, and 60 min. (B) The protein levels of IκB-α were determined by western blot analysis. (C) Statistical results of western blot analysis. Data are presented as the mean±SD of three independent experiments. ** P<0.01, *** P<0.001 vs RANKL‑treated control.

The activation of NF-κB induced by RANKL is crucial for osteoclast biology [37]. Therefore, NF-κB transcriptional activity was determined. BMMs were transfected with an NF-κB luciferase reporter construct and pretreated with SPN (0, 50, and 200 μM) for 30 min, followed by stimulation with 50 ng/mL RANKL for 6 h. The NF-κB luciferase reporter assay results showed that only treatment with RANKL increased the luciferase activity, and SPN significantly inhibited RANKL-induced NF-κB activity in a dose-dependent manner ( Figure 4A). Then, to explore the role of SPN on RANKL-mediated IκB-α expression, BMMs were pretreated with SPN (200 μM) for 30 min, and then treated with 50 ng/mL RANKL for different time (0 to 60 min). As shown in Figure 4B,C, SPN strongly suppresses IκB-α degradation, with the most significant reduction at 20 min.

### Specnuezhenide suppresses RANKL-induced MAPK activation

**Figure 5:**
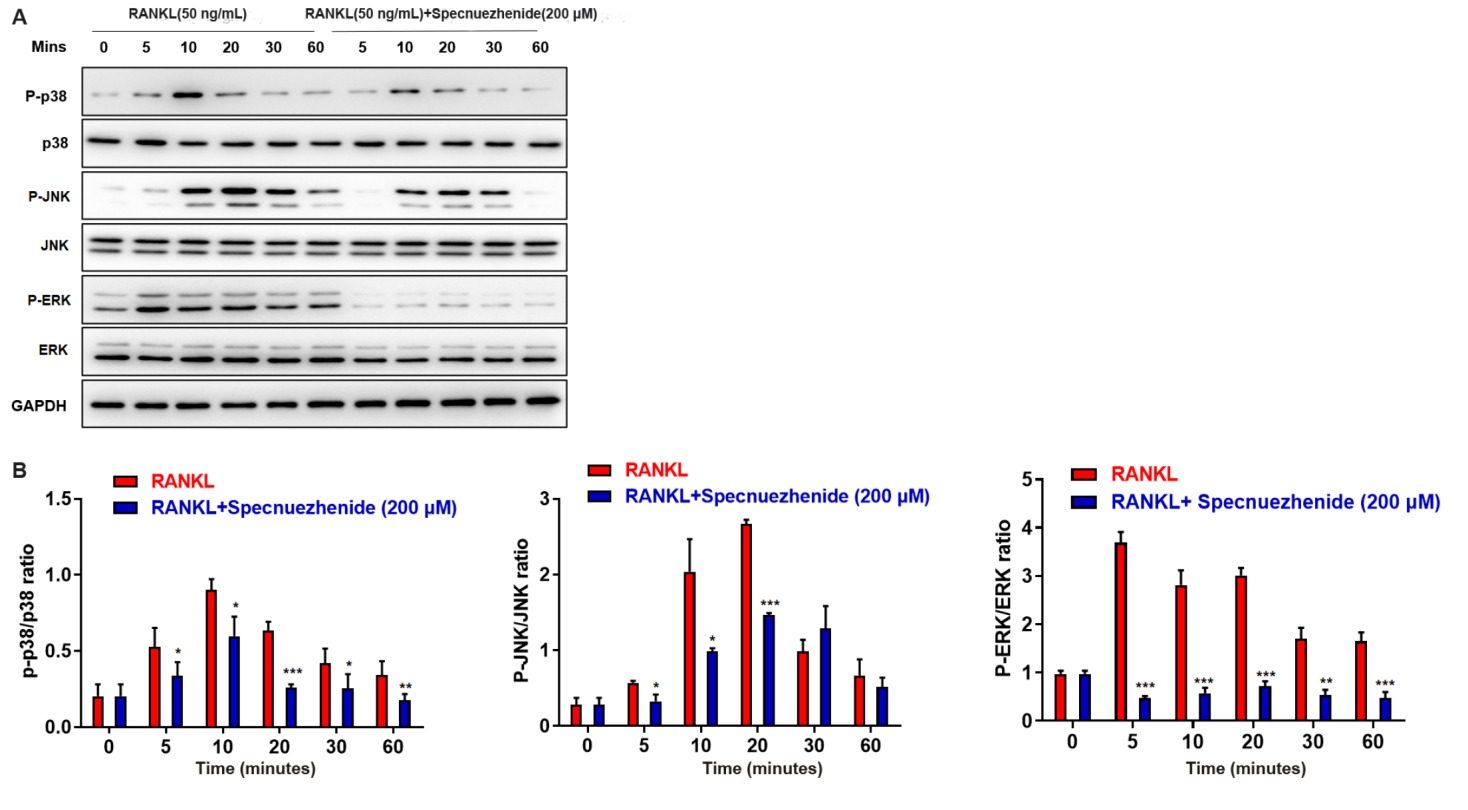
Figure 5 Specnuezhenide attenuates RANKL-induced MAPK activation BMMs were pretreated with 200 μM SPN, followed by RANKL (50 ng/mL) stimulation for the indicated time periods (0, 5, 10, 30, and 60 min). (A) The protein levels were determined by western blot analysis. (B) Statistical results of western blot analysis assay. Data are presented as the mean±SD of three independent experiments. * P<0.05, ** P<0.01, *** P<0.001 vs RANKL‑treated control.

RANKL can activate MAPK pathways during osteoclast formation [39]. To further explore the molecular mechanism of SPN inhibition of c-Fos and NFATc1 expression and activation of NF-κB, we observed the effects of SPN on RANKL-induced MAPK pathways. As shown in Figure 5A,B, RANKL stimulation led to peaks in the levels of p-p38/p38, p-JNK/JNK, and p-ERK/ERK. SPN significantly attenuated the levels of p-p38/p38, p-JNK/JNK, and p-ERK/ERK after 5 min, respectively ( Figure 5A,B). These results indicate that the mechanism by which SPN inhibits osteoclastogenesis involves the inhibition of the MAPK (ERK, p38, and JNK) signaling pathway.

### Specnuezhenide suppresses diabetes-induced bone loss

**Figure 6:**
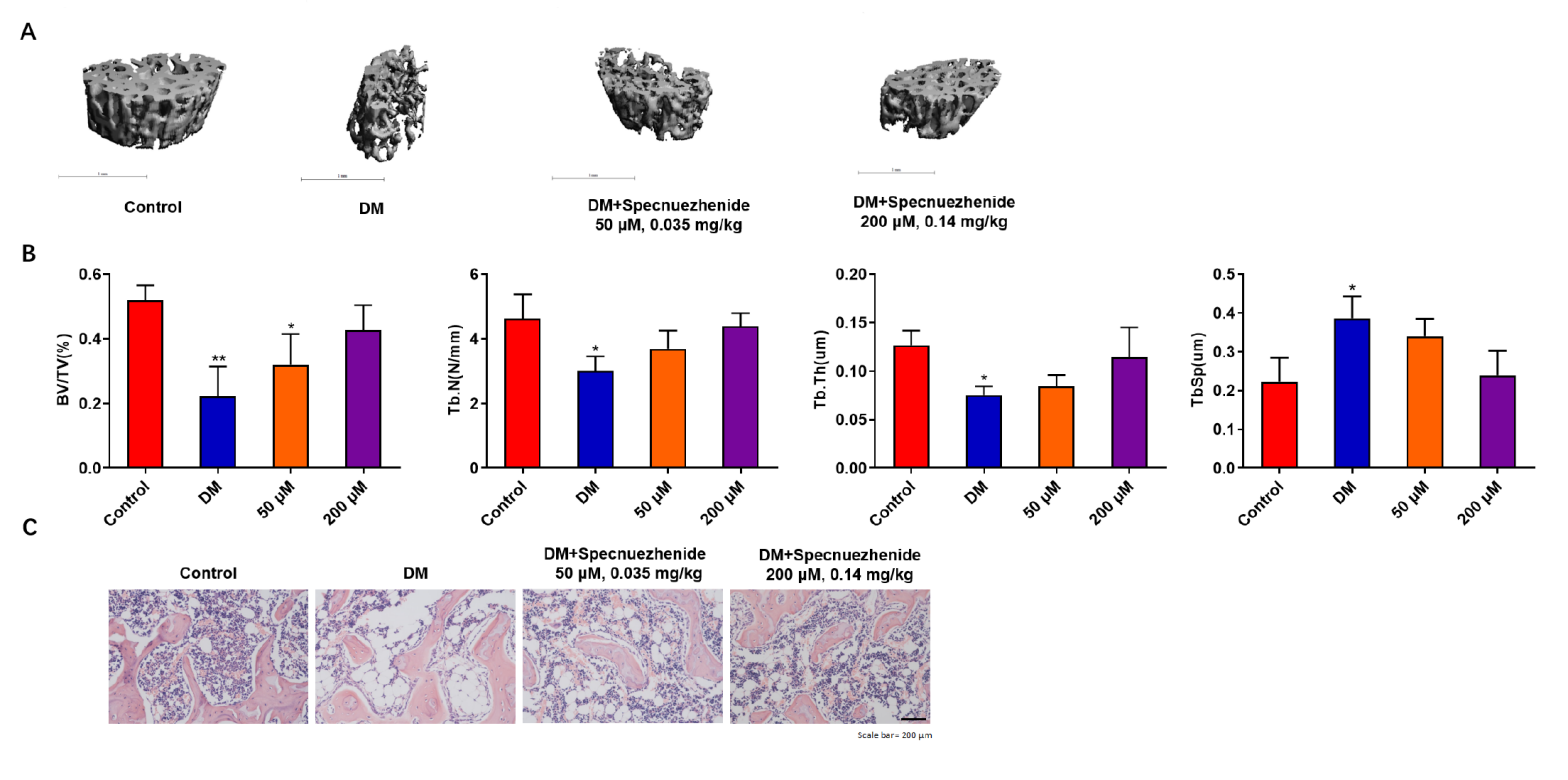
Figure 6 Specnuezhenide prevents type 2 diabetes-induced bone loss Rats were sacrificed to isolate the femurs for analysis after 6 weeks of treatment. (A) Three-dimensional reconstructed images of the femurs from the control, DM, DM+ SPN (0.035 mg/kg), and DM+SPN (0.14 mg/kg) groups (n=10) 8 days after treatment. (B) The femurs were subjected to micro-CT for bone evaluation. CT Analyzer software was used to evaluate BV/TV, Tb.N, Tb.Th and Tb.Sp (n=10 per group). (C) H&E staining analysis of the metaphyseal region of femoral bones from the control, DM, DM+SPN (0.035 mg/kg), and DM+SPN (0.14 mg/kg) groups. Scale bar= 200 μm. * P<0.05, ** P<0.01, *** P<0.001 vs RANKL-treated control.

Finally, we tested the effect of SPN in DM animal model. In the animal experiment, DM model rats were injected with SPN at doses of 50 μM or 200 μM. Serum was isolated from each rat for biochemical analysis. Compared with the control group, the model group showed increased levels of blood glucose, HbA1c, LDL-C, TC, and TG, and decreased levels of insulin and HDL-c, while the SPN-treated group exhibited opposite effects on these diabetes-related markers ( Table 2 and Table 3). In addition, 200 μM SPN treatment decreased serum bone turnover markers, such as osteocalcin, ALP, TRACP 5b, PINP, and CTX-1 ( Table 4). SPN also decreased RANKL level and increased RUNX2, OPG, Ca, and P levels ( Table 5). Micro-CT analysis showed that 200 μM SPN suppressed bone mass loss in the DM model, while 50 μM SPN had no effect on bone mass loss ( Figure 6A). Quantitative analysis confirmed that the 200 μM SPN-treatment group had increased bone parameters (BV/TV, Tb.N, Tb.Th), and decreased Tb.Sp compared with the model group ( Figure 6B). H&E staining further confirmed that diabetes-induced bone mass loss was significantly reduced in the 200 μM SPN-treatment group compared with the model group ( Figure 6C). These results indicate that SPN suppresses diabetes-induced bone loss.

**Table:** **Table 2** Specnuezhenide decreases glucose, HbA1c levels and increases insulin level

Parameter	Control	DM	DM +50 μM SPN	DM +200 μM SPN
Glucose (mg/dL)	89.14±0.34	150.83±2.40 ^***^	144.93±2.06	131.04±0.62 ^#^
Insulin (ng/mL)	4.29±0.40	0.81±0.09 ^***^	0.85±0.12	1.62±0.34 ^#^
HbA1c (%)	3.77±0.52	20.41±1.77 ^***^	18.76±1.46 ^#^	16.06±1.44 ^#^

*** *P*<0.001 vs Control; ^#^ *P*<0.05 vs DM.

**Table:** **Table 3** Specnuezhenide decreases serum levels of LDL-C, TC and TG and increases HDL-C level

Parameter	Control	DM	DM +50 μM SPN	DM +200 μM SPN
LDL-C (mM)	0.62±0.06	1.28±0.39 ^**^	1.07±0.24	0.94±0.31 ^#^
HDL-C (mM)	0.38±0.03	0.15±0.04 ^***^	0.19±0.02	0.29±0.07 ^#^
TC (mM)	1.09±0.10	5.53±0.60 ^***^	5.01±0.41	4.89±0.46 ^#^
TG (mM)	0.94±0.08	4.89±0.68 ^***^	4.67±0.53	4.45±0.42 ^#^

** *P*<0.01, *** *P*<0.001 vs Control; ^#^ *P*<0.05 vs DM.

**Table:** **Table 4** Specnuezhenide decreases serum levels of bone turnover markers

Parameter	Control	DM	DM +50 μM SPN	DM +200 μM SPN
Osteocalcin (ng/mL)	17.65±1.92	86.06±7.80 ^***^	80.62±9.24	36.01±3.07 ^##^
ALP (U/dL)	119.38±15.50	592±62.02 ^***^	524.20±56.28	212.40±24.92 ^##^
TRACP 5b (U/L)	1.90±0.16	9.57±1.46 ^***^	8.31±0.75	4.06±0.39 ^##^
PINP (μg/L)	35.93±3.91	198.14±21.19 ^***^	155.18±18.04	79.71±7.51 ^##^
CTX-1 (ng/mL)	16.92±1.53	88.95±8.79 ^***^	84.32±8.18	47.24±4.54 ^##^

*** *P*<0.001 vs Control; ^##^ *P*<0.01 vs DM.

**Table:** **Table 5** Specnuezhenide increases serum levels of RUNX 2, OPG, Ca, P and decreases serum RANKL level

Parameter	Control	DM	DM +50 μM SPN	DM +200 μM SPN
RUNX 2 (ng/mL)	11.48±1.35	2.13±0.16 ^***^	3.12±2.67	8.56±0.69 ^###^
RANKL (ng/mL)	2.48±0.29	12.50±1.28 ^***^	11.53±1.95	5.39±0.67 ^##^
OPG (ng/mL)	7.06±0.82	1.36±0.19 ^***^	2.17±0.29 ^#^	5.67±0.69 ^###^
Ca (mg/dL)	9.27±0.90	1.60±0.79 ^***^	2.94±0.37 ^#^	7.02±0.84 ^###^
P (mg/dL)	7.45±0.73	1.45±0.19 ^***^	2.28±0.25 ^#^	5.86±0.46 ^###^

*** *P*<0.001 vs Control; ^#^ *P*<0.05, ^##^ *P*<0.01, ^###^ *P*<0.001 vs DM.

## Discussion

With the increasing improvement of living standards, DM has become the most common endocrine and metabolic disorder threatening human health, accompanied by the occurrence of serious complications [40]. Among these disorders, diabetic osteoporosis has become a hot topic of discussion [41]. Diabetic osteoporosis is a systemic metabolic bone disease characterized by decreased bone mass and degeneration of bone microstructure [42]. Strotmeyer *et al*. [13] found that among patients with diabetes, approximately 50%–66% showed a decreasing trend in bone mineral density, and approximately 33% were diagnosed with osteoporosis. Moreover, studies revealed that compared with that in healthy individuals, the risk of fracture in diabetic patients is increased by 2–3 times, and the risk increases over time; when diabetes lasts for more than 15 years, the risk is 3 times greater than that in the healthy individuals [14]. Therefore, diabetic osteoporosis has become a particularly prominent worldwide topic seriously affecting human health. However, its pathogenesis is still unclear, affecting its effective prevention and treatment.

In recent years, natural compounds extracted from plants have attracted increasing attention as anti-bone resorption drugs. A series of natural compounds, such as luteoloside [43], aliseol-B [44], andrographolide [45], (+)-vitisin A [46], matairesinol [47] and quercetin [48], reportedly play an important role in the treatment of osteoclast-associated osteolytic diseases. Therefore, natural compounds may be a new treatment scheme for osteoclast-associated osteolytic diseases, and the study of the mechanism and application of natural compounds on the biological effects of osteoclasts is worthy of attention and further study. SPN was isolated from the fruit of *Ligustrum Ligustri*, and studies have shown that it plays an important role in the neural system and diabetic retinopathy [ 49, 50] . Ma *et al*. [51] found that SPN could inhibit chondrocyte inflammation by inhibiting the transmission of NF-κB and Wnt/β-catenin signals, thus playing an anti-inflammatory role in osteoarthritis. The role and regulatory mechanism of SPN in diabetic osteoporosis disease are still unclear. In the present study, we clarified the positive role of SPN in osteoclast formation. Osteoclasts are large multinucleated cells formed by macrophages that are responsible for bone absorption and release of mineral matrix and play an important role in the pathological destruction of bone. In this study, we investigated the effect of SPN on diabetic osteoporosis and its regulatory mechanism. First, the biological function of SPN was evaluated by osteoclast differentiation assay, and the results showed that SPN had an inhibitory effect on osteoclast formation *in vitro*. More importantly, the MTT assay showed that SPN inhibited osteoclast activity but did not affect cell activity at concentrations up to 200 μM. This result suggests that our study provides a relatively safe treatment option for osteoclast-associated diseases, as SPN does not cause nonspecific cytotoxicity or result in adverse side effects. Then, we detected the expression levels of osteoclast differentiation-related genes, consistent with the inhibitory effect of osteoclasts, and observed reduced expressions of osteoclast marker genes, such as *cathepsin K*, *TRAP*, and *calcitonin receptor*, indicating that the inhibitory effect of SPN on osteoclast formation is dose-dependent.

In addition, two key factors in osteoclast formation have been reported, i.e., c-Fos and NFATc1, which promote the expressions of downstream genes [ 52, 53] . c-Fos induces NFATc1 to modulate osteoclast formation after RANKL stimulation [54]. Consistent with previous results, we found that SPN significantly reduced NFATc1 and c-Fos protein expressions during osteoclast differentiation by western blot analysis. RANKL-induced upregulation of c-Fos and NFATc1 mRNA and protein was dramatically downregulated by SPN pretreatment, indicating that the c-Fos/NFATc1 pathway is a target of the inhibitory effect of SPN on osteoclast differentiation. The inhibitory effect of SPN on the expressions of osteoclast-specific genes suggests that SPN may inhibit some signaling pathways of osteoclast differentiation.

The combination of NFATc1 and other transcription factors further activates osteoclast-specific genes, further explaining the previous assayed results. RANKL binding to its receptor (RANK) on osteoclast precursors leads to a cascade of intracellular events, including NF-κB, AKT, MAPKs, NFAT, ionic calcium and calcium/calmodulin-dependent kinase. Among these signaling pathways, NF-κB and MAPKs are two major pathways related to osteoclastogenesis [ 55, 56] . RANKL-induced NF-κB activation occurs early in osteoclast differentiation and induces the expressions of osteoclast-associated genes [ 57, 58] . RANKL binding causes the binding of its receptor RANK to TRAF6, forming a complex to activate downstream TAKl and induce IKKα [59]. Activated IKKα regulates the degradation of IκBα. NF-κB is released through the degradation of IκBα and then translocates from the cytoplasm to the nucleus, and subsequently activates osteoclastogenesis gene transcription directly to modulate osteoclast formation and function [ 60– 62] . Zhou *et al*. [61] found that NF-κB activity and IκBα protein degradation were inhibited by berberine sulfate. In addition, NLRP12 serves as a negative regulator of inflammation and osteoclast formation via inhibition of IκB-α degradation and downregulation of the NF-κB pathway [62]. Therefore, we investigated the effects of SPN on the NF-κB pathway by detecting NF-κB transcriptional activity and IκB-α protein expression. We found that SPN inhibited RANKL-induced NF-κB activity and prevented IκB-α degradation, indicating that SPN suppressed RANKL-induced NF-κB activation. Studies have shown that MAPKs play a key role in the regulation of bone formation and bone homeostasis, especially in the differentiation of osteoblasts and osteoclasts [63]. ERK, JNK and p38 belong to the traditional MAPK family kinases [64]. At the early stage of osteoclast differentiation, RANKL can induce the temporary activation of the MAPK signaling pathway. Moreover, downregulation of triggering receptors expressed on myeloid cells 2 (TREM-2) expression can weaken the activation of the RANKL-induced calmodulin-dependent protein kinases (CaMKs)-MEK-ERK signaling pathway and reduce the expression of NFATc1 [65]. Therefore, we investigated the effects of SPN on the MAPK pathway by detecting p-ERK, ERK, p-p38, p38, p-JNK, and JNK expressions. We found that SPN significantly attenuated p-ERK/ERK, p-p38/p38, and p-JNK/JNK expressions. In general, we conclude that SPN inhibits the activation of RANKL-mediated NF-κB and MAPK signaling pathways at the cellular level in the early stage of osteoclast formation, which inhibits the activation of downstream factors, such as NFATc1 and c-Fos.

We further tested the role of SPN *in vivo* using a diabetic rat model. Consistent with the *in vitro* results, our *in vivo* study showed that SPN prevented bone loss and osteoclast formation. Moreover, we analyzed the role of SPN on diabetes. Interestingly, SPN decreased the serum glucose, HbA1c, LDL-C, TC and TG levels and effectively improved the insulin and HDL-C levels. These results suggested that SPN had a protective role in DM. In addition, a previous study reported that SPN could regulate blood sugar control and glucose tolerance in gestational diabetic rats [24]. These findings suggest that SPN suppresses diabetes-induced bone loss by inhibiting RANKL-induced osteoclastogenesis. However, this study still has some limitations. For example, in the present study, only a part of cancellous bone in femur was extracted to perform 3D reconstructions and H&E staining. In addition, further work is required to determine whether SPN inhibits osteoclast formation and bone loss through the NF-κB and MAPK pathways.

In summary, we found that SPN has an inhibitory effect on the osteoclast formation of BMMs, suggesting that SPN may have anti-osteoclast and anti-bone resorption activities. We further elucidated the molecular mechanism of the SPN effects, including inhibition of NF-κB activation and the levels of downstream factors c-Fos and NFATc1, and inhibition of JNK and p38 phosphorylation. Therefore, we believe that SPN may help treat osteoclast-related bone diseases, although further studies are needed to determine the appropriate dose and treatment strategy. SPN may also be used as a potential traditional Chinese medicine to prevent and treat diabetes-induced osteoporosis disease. In the era of precision medicine, accurate targeting and appropriate drug concentrations are the keys for using natural compounds to treat osteoclast-related osteolytic diseases in the future.

## Supplementary Data

Supplementary data is available at *Acta Biochimica et Biophysica Sinica* online.

## References

[1] Poiana C, Capatina C (2017). Fracture risk assessment in patients with diabetes mellitus. J Clin Densitometry.

[2] Shu L, Beier E, Sheu T, Zhang H, Zuscik MJ, Puzas EJ, Boyce BF (2015). High-fat diet causes bone loss in young mice by promoting osteoclastogenesis through alteration of the bone marrow environment. Calcif Tissue Int.

[3] Stabley JN, Prisby RD, Behnke BJ, Delp MD (2015). Type 2 diabetes alters bone and marrow blood flow and vascular control mechanisms in the ZDF rat. J Endocrinol.

[4] Lecka-Czernik B, Stechschulte LA, Czernik PJ, Dowling AR (2015). High bone mass in adult mice with diet-induced obesity results from a combination of initial increase in bone mass followed by attenuation in bone formation; implications for high bone mass and decreased bone quality in obesity. Mol Cell Endocrinol.

[5] Yu W, Zhong L, Yao L, Wei Y, Gui T, Li Z, Kim H (2021). Bone marrow adipogenic lineage precursors promote osteoclastogenesis in bone remodeling and pathologic bone loss. J Clin Investigation.

[6] Teitelbaum SL (2000). Bone resorption by osteoclasts. Science.

[7] Park-Min KH (2018). Mechanisms involved in normal and pathological osteoclastogenesis. Cell Mol Life Sci.

[8] Stapleton M, Sawamoto K, Alméciga-Díaz CJ, Mackenzie WG, Mason RW, Orii T, Tomatsu S (2017). Development of bone targeting drugs. Int J Mol Sci.

[9] Lacey DL, Timms E, Tan HL, Kelley MJ, Dunstan CR, Burgess T, Elliott R (1998). Osteoprotegerin ligand is a cytokine that regulates osteoclast differentiation and activation. Cell.

[10] Kong YY, Yoshida H, Sarosi I, Tan HL, Timms E, Capparelli C, Morony S (1999). OPGL is a key regulator of osteoclastogenesis, lymphocyte development and lymph-node organogenesis. Nature.

[11] Li J, Sarosi I, Yan XQ, Morony S, Capparelli C, Tan HL, McCabe S (2000). RANK is the intrinsic hematopoietic cell surface receptor that controls osteoclastogenesis and regulation of bone mass and calcium metabolism. Proc Natl Acad Sci USA.

[12] Lee SE, Woo KM, Kim SY, Kim HM, Kwack K, Lee ZH, Kim HH (2002). The phosphatidylinositol 3-kinase, p38, and extracellular signal-regulated kinase pathways are involved in osteoclast differentiation. Bone.

[13] Lee JH, Jin H, Shim HE, Kim HN, Ha H, Lee ZH (2010). Epigallocatechin-3-gallate Inhibits osteoclastogenesis by down-regulating c-Fos expression and suppressing the nuclear factor-κB signal. Mol Pharmacol.

[14] Wu C, Wang W, Tian B, Liu X, Qu X, Zhai Z, Li H (2015). Myricetin prevents titanium particle-induced osteolysis
*in vivo* and inhibits RANKL-induced osteoclastogenesis
*in vitro*. Biochem Pharmacol.

[15] Thummuri D, Jeengar MK, Shrivastava S, Nemani H, Ramavat RN, Chaudhari P, Naidu VGM (2015). Thymoquinone prevents RANKL-induced osteoclastogenesis activation and osteolysis in an in vivo model of inflammation by suppressing NF-KB and MAPK Signalling. Pharmacol Res.

[16] Vattakuzhi Y, Abraham SM, Freidin A, Clark AR, Horwood NJ (2012). Dual-specificity phosphatase 1-null mice exhibit spontaneous osteolytic disease and enhanced inflammatory osteolysis in experimental arthritis. Arthritis Rheumatism.

[17] Thouverey C, Caverzasio J (2012). The p38α MAPK positively regulates osteoblast function and postnatal bone acquisition. Cell Mol Life Sci.

[18] Thummuri D, Naidu VGM, Chaudhari P (2017). Carnosic acid attenuates RANKL-induced oxidative stress and osteoclastogenesis via induction of Nrf2 and suppression of NF-κB and MAPK signalling. J Mol Med.

[19] Sigal LH (2006). Basic science for the clinician 39. J Clin Rheumatol.

[20] Liu C, Ma R, Wang L, Zhu R, Liu H, Guo Y, Zhao B (2017). Rehmanniae Radix in osteoporosis: A review of traditional Chinese medicinal uses, phytochemistry, pharmacokinetics and pharmacology. J EthnoPharmacol.

[21] Guo N, Yu Y, Ablajan K, Li L, Fan B, Peng J, Yan H (2011). Seasonal variations in metabolite profiling of the fruits of
*Ligustrum lucidum* Ait. Rapid Commun Mass Spectrom.

[22] Wu J, Ke X, Fu W, Gao X, Zhang H, Wang W, Ma N (2016). Inhibition of hypoxia-induced retinal angiogenesis by specnuezhenide, an effective constituent of Ligustrum lucidum Ait., through suppression of the HIF-1α/VEGF signaling pathway. Molecules.

[23] Fu G, Ip FCF, Pang H, Ip NY (2010). New secoiridoid glucosides from
*ligustrum lucidum* induce ERK and CREB phosphorylation in cultured cortical neurons. Planta Med.

[24] Yang J, Jia J, Yang Y, Zhao Y, Li Q (2020). Protective effect of Specnuezhenide on islet Î² cell of rats with gestational diabetes mellitus. Cell Mol Biol (Noisy-le-grand).

[25] Wang-Fischer Y, Garyantes T (2018). Improving the reliability and utility of streptozotocin-induced rat diabetic model. J Diabetes Res.

[26] Zhao YQ, Liu YQ, Yuan JF, Dai X, Niu MM, Sun XM, Kuang DX (2018). Regeneration of islet β-cells in tree shrews and rats. Animal Model Exp Med.

[27] Magalhães DAD, Kume WT, Correia FS, Queiroz TS, Allebrandt Neto EW, Santos MPD, Kawashita NH (2019). High-fat diet and streptozotocin in the induction of type 2 diabetes mellitus: a new proposal. An Acad Bras Ciênc.

[28] Gheibi S, Kashfi K, Ghasemi A (2017). A practical guide for induction of type-2 diabetes in rat: Incorporating a high-fat diet and streptozotocin. Biomed Pharmaco Ther.

[29] Yoon JY, Baek CW, Kim HJ, Kim EJ, Byeon GJ, Yoon JU (2018). Remifentanil negatively regulates RANKL-induced osteoclast differentiation and bone resorption by inhibiting c-Fos/NFATc1 expression. Tissue Eng Regen Med.

[30] Du J, Cheng B, Zhu X, Ling C (2011). Ginsenoside Rg1, a novel glucocorticoid receptor agonist of plant origin, maintains glucocorticoid efficacy with reduced side effects. J Immunol.

[31] Wang C, Steer JH, Joyce DA, Yip KHM, Zheng MH, Xu J (2003). 12-
*O*-tetradecanoylphorbol-13-acetate (TPA) Inhibits Osteoclastogenesis by Suppressing RANKL-Induced NF-κB Activation. J Bone Miner Res.

[32] Wilches-Buitrago L, Viacava PR, Cunha FQ, Alves-Filho JC, Fukada SY (2019). Fructose 1,6-bisphosphate inhibits osteoclastogenesis by attenuating RANKL-induced NF-κB/NFATc-1. Inflamm Res.

[33] Takayanagi H, Kim S, Koga T, Nishina H, Isshiki M, Yoshida H, Saiura A (2002). Induction and activation of the transcription factor NFATc1 (NFAT2) integrate RANKL signaling in terminal differentiation of osteoclasts. Dev Cell.

[34] Feng HT, Cheng T, Steer JH, Joyce DA, Pavlos NJ, Leong CL, Kular J (2009). Myocyte enhancer factor 2 and microphthalmia-associated transcription factor cooperate with NFATc1 to transactivate the V-ATPase d2 promoter during RANKL-induced osteoclastogenesis. J Biol Chem.

[35] Ortega N, Behonick D, Stickens D, Werb Z (2003). How proteases regulate bone morphogenesis. Ann New York Acad Sci.

[36] Delaissé JM, Engsig MT, Everts V, del Carmen Ovejero M, Ferreras M, Lund L, Vu TH (2000). Proteinases in bone resorption: obvious and less obvious roles. Clinica Chim Acta.

[37] Takayanagi H (2007). Osteoimmunology: shared mechanisms and crosstalk between the immune and bone systems. Nat Rev Immunol.

[38] Asagiri M, Takayanagi H (2007). The molecular understanding of osteoclast differentiation. Bone.

[39] Choi JH, Han Y, Kim YA, Jin SW, Lee GH, Jeong HM, Lee HS (2017). Platycodin D inhibits osteoclastogenesis by repressing the NFATc1 and MAPK signaling pathway. J Cell Biochem.

[40] Kumar S, Behl T, Sachdeva M, Sehgal A, Kumari S, Kumar A, Kaur G (2021). Implicating the effect of ketogenic diet as a preventive measure to obesity and diabetes mellitus. Life Sci.

[41] Ma H, Wang X, Zhang W, Li H, Zhao W, Sun J, Yang M (2020). Melatonin suppresses ferroptosis induced by high glucose via activation of the Nrf2/HO-1 signaling pathway in type 2 diabetic osteoporosis. Oxid Med Cell Longev.

[42] Tafaro L, Napoli N. Current and Emerging Treatment of Osteoporosis. In: Falaschi P, Marsh D eds. Orthogeriatrics: The Management of Older Patients with Fragility Fractures Cham (CH): Springer Copyright 2021, The Author(s). 2021: 257–272.

[43] Song F, Wei C, Zhou L, Qin A, Yang M, Tickner J, Huang Y (2018). Luteoloside prevents lipopolysaccharide‐induced osteolysis and suppresses RANKL‐induced osteoclastogenesis through attenuating RANKL signaling cascades. J Cell Physiol.

[44] Lee JW, Kobayashi Y, Nakamichi Y, Udagawa N, Takahashi N, Im NK, Seo HJ (2010). Alisol-B, a novel phyto-steroid, suppresses the RANKL-induced osteoclast formation and prevents bone loss in mice. Biochem Pharmacol.

[45] Wang T, Liu Q, Zhou L, Yuan JB, Lin X, Zeng R, Liang X (2015). Andrographolide inhibits ovariectomy-induced bone loss via the suppression of RANKL signaling pathways. Int J Mol Sci.

[46] Chiou WF, Huang YL, Liu YW (2014). (+)-Vitisin A inhibits osteoclast differentiation by preventing TRAF6 ubiquitination and TRAF6-TAK1 formation to suppress NFATc1 activation. PLoS ONE.

[47] Choi SW, Park KI, Yeon JT, Ryu BJ, Kim KJ, Kim SH (2014). Anti-osteoclastogenic activity of matairesinol via suppression of p38/ERK-NFATc1 signaling axis. BMC Complement Altern Med.

[48] Satué M, Arriero MM, Monjo M, Ramis JM (2013). Quercitrin and taxifolin stimulate osteoblast differentiation in MC3T3-E1 cells and inhibit osteoclastogenesis in RAW 264.7 cells. Biochem Pharmacol.

[49] Kawaguchi H (2009). Regulation of osteoarthritis development by wnt–β-catenin signaling through the endochondral ossification process. J Bone Mineral Res.

[50] Cheleschi S, De Palma A, Pecorelli A, Pascarelli NA, Valacchi G, Belmonte G, Carta S (2017). Hydrostatic pressure regulates microRNA expression levels in osteoarthritic chondrocyte cultures via the Wnt/β-Catenin pathway. Int J Mol Sci.

[51] Ma C, Zhou X, Xu K, Wang L, Yang Y, Wang W, Liu A (2018). Specnuezhenide decreases interleukin-1β-induced inflammation in rat chondrocytes and reduces joint destruction in osteoarthritic rats. Front Pharmacol.

[52] Deepak V, Kruger MC, Joubert A, Coetzee M (2015). Piperine alleviates osteoclast formation through the p38/c-Fos/NFATc1 signaling axis. BioFactors.

[53] Kim JH, Kim M, Jung HS, Sohn Y (2019). *Leonurus sibiricus* L. ethanol extract promotes osteoblast differentiation and inhibits osteoclast formation. Int J Mol Med.

[54] Kim MH, Lee H, Ha IJ, Yang WM (2021). Zanthoxylum piperitum alleviates the bone loss in osteoporosis via inhibition of RANKL-induced c-fos/NFATc1/NF-κB pathway. Phytomedicine.

[55] David JP, Sabapathy K, Hoffmann O, Idarraga MH, Wagner EF (2002). JNK1 modulates osteoclastogenesis through both c-Jun phosphorylation-dependent and -independent mechanisms. J Cell Sci.

[56] Boyce BF, Xing L, Franzoso G, Siebenlist U (1999). Required and nonessential functions of nuclear factor-kappa B in bone cells. Bone.

[57] Yun J, Lee KY, Park B (2019). Neotuberostemonine inhibits osteoclastogenesis via blockade of NF-κB pathway. Biochimie.

[58] Xiao L, Zhong M, Huang Y, Zhu J, Tang W, Li D, Shi J (2020). Puerarin alleviates osteoporosis in the ovariectomy-induced mice by suppressing osteoclastogenesis via inhibition of TRAF6/ROS-dependent MAPK/NF-κB signaling pathways. Aging.

[59] Mulero MC, Huxford T, Ghosh G. NF-κB, IκB, and IKK: Integral components of immune system signaling.
*
Adv Exp Med Biol
* 2019, 1172: 207–226. https://doi.org/10.1007/978-981-13-9367-9_10.

[60] Zhou L, Liu Q, Yang M, Wang T, Yao J, Cheng J, Yuan J (2016). Dihydroartemisinin, an anti-malaria drug, suppresses estrogen deficiency-induced osteoporosis, osteoclast formation, and RANKL-induced signaling pathways. J Bone Miner Res.

[61] Zhou L, Song F, Liu Q, Yang M, Zhao J, Tan R, Xu J (2015). Berberine sulfate attenuates osteoclast differentiation through RANKL induced NF-κB and NFAT pathways. Int J Mol Sci.

[62] Taira TM, Lima V, Prado DS, Silva TA, Issa JPM, da Silva LAB, Zamboni DS (2019). NLRP12 attenuates inflammatory bone loss in experimental apical periodontitis. J Dent Res.

[63] Fang C, He M, Li D, Xu Q (2021). YTHDF2 mediates LPS-induced osteoclastogenesis and inflammatory response via the NF-κB and MAPK signaling pathways. Cell Signalling.

[64] Decean HP, Brie IC, Tatomir CB, Perde-Schrepler M, Fischer-Fodor E, Virag P (2018). Targeting MAPK (p38, ERK, JNK) and inflammatory CK (GDF-15, GM-CSF) in UVB-Activated Human Skin Cells with Vitis vinifera Seed Extract. J Environ Pathol Toxicol Oncol.

[65] Chang JH, Chang EJ, Kim HH, Kim SK (2009). Enhanced inhibitory effects of a novel CpG motif on osteoclast differentiation via TREM-2 down-regulation. Biochem Biophys Res Commun.

